# *Drosophila* as a Model to Study Brain Innate Immunity in Health and Disease

**DOI:** 10.3390/ijms19123922

**Published:** 2018-12-07

**Authors:** Shu Hui Lye, Stanislava Chtarbanova

**Affiliations:** Department of Biological Sciences, 300 Hackberry lane, Tuscaloosa, AL 35487, USA; slye@crimson.ua.edu

**Keywords:** *Drosophila*, innate immunity, inflammaging, neurodegeneration, brain infection, phagocytosis, autophagy

## Abstract

Innate immunity is the first line of defense against invading pathogens and plays an essential role in defending the brain against infection, injury, and disease. It is currently well recognized that central nervous system (CNS) infections can result in long-lasting neurological sequelae and that innate immune and inflammatory reactions are highly implicated in the pathogenesis of neurodegeneration. Due to the conservation of the mechanisms that govern neural development and innate immune activation from flies to mammals, the lack of a classical adaptive immune system and the availability of numerous genetic and genomic tools, the fruit fly *Drosophila melanogaster* presents opportunities to investigate the cellular and molecular mechanisms associated with immune function in brain tissue and how they relate to infection, injury and neurodegenerative diseases. Here, we present an overview of currently identified innate immune mechanisms specific to the adult *Drosophila* brain.

## 1. Introduction

Innate immunity represents the first line of defense against microbial invaders and plays a significant role in limiting pathogen spread as well as in activating the long-lasting adaptive immunity [1,2]. Inflammation, a key aspect of immunity, is a central process that responds to tissue injury and infection and is orchestrated by various immune cell types and cytokines [3]. Acute inflammation is a beneficial process that contributes to the containment and eradication of infection threats, whereas prolonged inflammatory responses can lead to significant tissue damage [3].

Within the central nervous system (CNS), infections are a source of significant mortality worldwide and can lead to long-lasting neurological sequelae in surviving individuals [4,5]. These infections can be caused by a plethora of pathogens, including bacteria and viruses, and can lead to acute manifestations such as meningitis [6] and encephalitis [7,8], or chronic neurodegenerative conditions such as Alzheimer’s disease [9,10]. Bacterial brain infections may represent an underlying condition in the development of psychiatric disorders such as schizophrenia and depression [11]. Infections with emerging neurotropic pathogens such as Zika virus (ZIKV) result in severe brain defects in newborns and neurological complications in adults [12]. While the brain was considered an immune privileged tissue, it is now clear that innate immune reactions also take place in the CNS to restrict pathogen replication and to induce antimicrobial protection [5,13]. For instance, protection against neurotropic viruses that replicate in the CNS relies on local innate immune responses mediated by the activation of Interferon (IFN) regulatory factors (IRFs) and nuclear factor kappa-light-chain-enhancer of activated B cells (NF-κB) signaling cascades and their downstream effectors [14]. These responses, which are mounted by neural cell types including microglia, astrocytes and neurons, use pattern recognition receptors (PRRs) to detect pathogen invasion and induce protective neuroinflammation [5,14]. However, the absence of proper resolution of neuroinflammation resulting in the failure to restore tissue homeostasis can result in pathological consequences and potential neurotoxicity [5].

A biological phenomenon called “inflammaging” corresponds to the age-dependent increase in the body’s pro-inflammatory status [15,16]. Inflammaging is characterized by elevated levels of pro-inflammatory cytokines such as Tumor Necrosis Factor-alpha (TNF-α) and Interleukine (IL)-6, which are also found up regulated in a NF-κB-dependent manner during aging in brain tissue [17,18]. Chronic inflammation in the CNS is becoming increasingly identified as an underlying mechanism of many neurological conditions including neurodegenerative diseases such as Alzheimer’s disease and Parkinson’s disease, as well as traumatic brain injury, spinal cord injury and stroke [19,20]. Moreover, inflammaging is considered a significant risk factor in neurodegenerative disease pathogenesis and progression [21,22].

The fruit fly *Drosophila melanogaster* is a widely appreciated model organism for studying fundamental biological processes, including the activation of innate immunity following infections with pathogens such as bacteria, fungi, parasites and viruses [23,24,25]. For example, the discovery of the critical role of the *Drosophila* Toll receptor in innate immunity activation [26] and the subsequent characterization of Toll-like receptors in mammals [27] illustrates the importance of this invertebrate model in deciphering critical biological pathways. More recently, flies have been used as a valuable experimental system to expand our understanding of innate antiviral defenses, and to identify novel genes and cellular pathways involved in resistance to viral infections [24,28,29,30,31]. Additionally, flies serve as a great model for investigating epithelial immune defenses and host–microbiota interactions, providing insights into innate immune mechanisms in barrier epithelia [32,33,34].

Comparative studies of brain development in vertebrates and invertebrates have shown remarkable similarities in gene expression, neural proliferation and brain circuit formation [35,36]. Therefore, the ability to study and model brain immune reactions in *Drosophila* could provide valuable insights into the molecular mechanisms associated with brain injury, infection, and neurodegenerative disease. In this review we summarize recent studies of innate immunity with focus on brain immune reactions, how they relate to pathological conditions such as neurodegeneration, but also neuroprotection in the context of aging, brain injury, and infection.

## 2. Innate Immune Reactions and Pathways in the Brain

In general, immune reactions in *Drosophila* can be categorized into systemic, epithelial and cellular immunity. The systemic immune response is characterized by the synthesis of immune effector molecules such as the antimicrobial peptides (AMPs) by the cells of the fat body—a functional equivalent of the mammalian liver—and their release into hemolymph—a fluid in insects analogous to blood—to address infections by microorganisms [25]. Epithelial immunity fights against invading microorganisms at the level of the barrier epithelia such as gut and trachea, and significantly contributes to the protection of flies [33]. For instance, in the gut, both the synthesis of AMPs [37,38] and production of reactive oxygen species (ROS) [39] characterize this response. The cellular immune response is centered on the action of hemocytes—the insect blood cells—, which play a major role in the phagocytosis of microorganisms and apoptotic cells [40,41,42]. In addition to these three types of immunity, proteolytic cascades contribute to melanization and coagulation reactions following wounding [43] and RNA interference (RNAi), inducible responses, as well as intrinsic immunity based on the action of restriction factors protect against virus infection [30].

As noted above, the brain was long considered to be an immune-privileged organ but has become of a particular interest for immunologists in recent years as the concept of immune privilege has been revisited [44]. Numerous studies have reported that the immune system also plays an important role in brain injury and neurodegenerative disease [19,45]. Like the mammalian brain, the fly CNS is isolated from the rest of the body and is protected from the potassium-rich hemolymph by the blood–brain barrier (BBB), warranting optimal neuronal function [46,47,48].

Because the mechanisms of neural development are conserved from flies to mammals, neurodegeneration and human neurodegenerative diseases can be effectively modeled in the fly [21,49]. Earlier work on fly models of axonal injury and more recently in the contexts of aging, neurodegeneration has attracted attention to the fly and suggested the use of *Drosophila* as a model of CNS immunity [50,51,52,53,54,55].

### 2.1. Inducible Response in the Brain

#### 2.1.1. NF-κB Signaling Pathways

In *Drosophila*, systemic infection with bacteria and fungi triggers the synthesis of AMPs, which is mediated by the evolutionary conserved NF-κB pathways Toll and immune deficiency (IMD). The Toll pathway is primarily activated in response to Gram-positive bacteria and fungi, whereas the IMD pathway is primarily activated in response to Gram-negative bacteria [25]. Activation of both pathways relies on the recognition of microbial cell wall components or virulence factors by *Drosophila* pattern recognition receptors (PRRs), leading to the nuclear translocation of NF-kB transcription factors and resulting in the expression of several hundreds of genes [56].

The cell wall of Gram-positive bacteria contains polymeric Lysine-type peptidoglycan (Lys-PGN), which is sensed by the soluble PRRs Peptidoglycan recognition protein SA (PGRP-SA) and Gram-negative bacteria binding protein 1 (GNBP1) leading to Toll pathway activation [57,58]. Fungal beta-glucans (β-glucans) are sensed by the soluble receptor GNBP3 and also induce the Toll pathway [59]. Sensing of microbial patterns is followed by the initiation of a proteolytic cascade in the hemolymph, triggering activation of the Toll receptor ligand Spaetzle via Spaetzle processing enzyme (SPE) and its subsequent binding to the Toll receptor [60,61]. Bacterial or fungal proteases that act as virulence factors are also able to activate SPE and the Toll pathway [59,62]. This leads to the nuclear translocation of the NF-κB transcription factors Dif and Dorsal leading to the expression of a group of antimicrobial peptides including Drosomycin.

**Figure 1:**
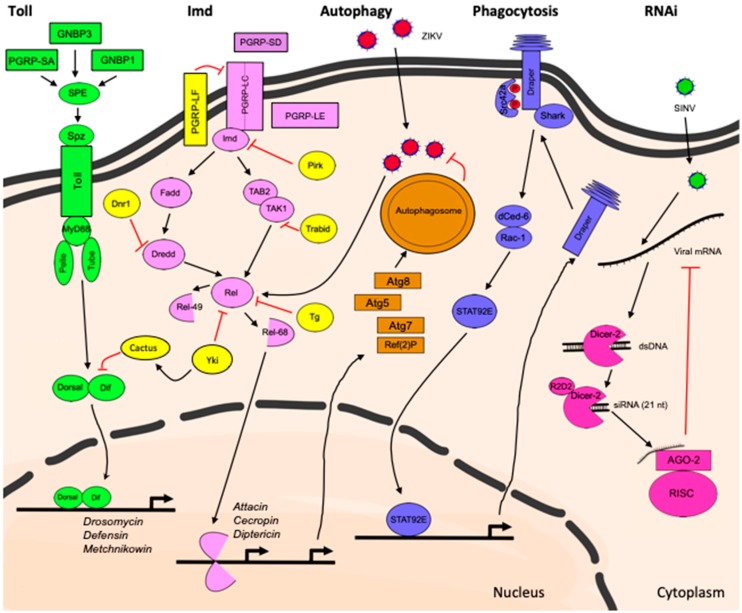
Innate immune pathways activated in the *Drosophila* brain following injury, infection and neurodegenerative disease.

The immune deficiency (IMD) pathway on the other hand is preferentially activated by sensing diaminopimelic acid-type peptidoglycan (DAP-PGN) [63], which is a component of the cell wall of Gram-negative bacteria, and regulates the expression of antibacterial peptides such as Diptericin following nuclear translocation of another NF-kB transcription factor: Relish [56]. Microbial-derived DAP-PGN directly binds to soluble, surface, and intracellular receptors such as PGRP-SD [64,65], PGRP-LC, and PGRP-LE [66], respectively, leading to activation of the IMD pathway. The membrane-associated protein PGRP-LF acts as a negative regulator of the IMD pathway by preventing its constitutive activation in the fly [67,68]. The components involved in Toll and IMD signaling cascades are shown in Figure 1.

##### Toll Pathway

A study in a *Drosophila* model of Alzheimer’s disease (AD) in which the major human peptide associated with AD pathology, amyloid-beta 42 (Aβ42), was overexpressed in photoreceptor cells in the eye showed that components of the Toll pathway mediate Aβ42-induced neurotoxicity [69]. In this model, the authors demonstrated that reducing the activity of the genes encoding the receptor Toll, the adaptor protein Tube, the kinase Pelle, and the transcription factors Dif and Dorsal could dominantly suppress the effects of Aβ42 overexpression, which manifests through a rough eye phenotype. Reduction in expression of components of the IMD pathway such as the adaptor protein Imd or the transcription factor Relish, however, did not suppress neurodegeneration in this model. Although brain expression of the transcription factor Dif and its inhibitor, the inhibitor of κB (IκB) ortholog Cactus, has been previously reported in *Drosophila* larvae [70], the brain-specific requirement of the Toll pathway in neurodegeneration following photoreceptor overexpression of Aβ42 has not been determined.

Additional lines of evidence implicate components of the Toll pathway in the nervous system’s immune reactions. In a fly model of amyotrophic lateral sclerosis (ALS), a devastating and rapidly progressing neurodegenerative condition, motorneuron-specific overexpression of the ALS-related RNA-binding protein 43-kDa TAR DNA-binding protein (TDP-43) leads to dose-dependent increase in expression of Toll pathway-related AMP genes including *drosomycin* and *defensin* in head tissue [71]. Moreover, the removal of one copy of the genes encoding the Toll receptor ligand Spz, the receptor Toll and the downstream transcription factor Dif reduces TDP-43-related neurotoxicity by improving lifespan and associated motility defects. Reduction of IMD signaling by omitting one copy of *relish* improves the lifespan but not the defective locomotion in this model, and the improvement observed is to a lesser extent than when mediated by components of the Toll pathway [71].

The AMP gene *metchnikowin*, which is another target of the Toll pathway during the systemic immune response, is induced in a fly model of CAG-repeat RNA-dependent neurodegeneration [72]. *metchnikowin* and *spz* are also found upregulated in fly heads following infliction of closed head traumatic brain injury (TBI) [73,74]. Although TBI leads to differential expression of genes that are targets of other innate immune pathways such as IMD and JAK/STAT [73,74], this study suggests that the *Drosophila* brain can respond to injury by activating the Toll pathway. Similarly, exposure of late third instar larvae to a 40-Gy dose of radiation leads to upregulation of Toll pathway target genes in the brains of surviving adults. *spz*, but not *relish* mutants are more sensitive to 30-Gy and 40-Gy dose of radiation, suggesting that the Toll pathway also plays a predominant role over the IMD pathway in radioprotection of the brain [75]. How exactly Spz is processed and whether its activation requires canonical upstream components of the Toll pathway such as SPE or pattern recognition receptors such as PGRP-SA, GNBP1, or GNBP3 in the contexts of TBI, radiation exposure and neurodegenerative disease is not known. SPE-independent activation of Spz has previously been reported in *Drosophila* [76], and might be relevant to Toll pathway activation in the brain. Known differences in the expression of Toll- and IMD-dependent antimicrobial peptide genes exist between the systemic immune response and activation of immunity in barrier epithelia such as the digestive tract and trachea [37,38]. In fact, AMP gene expression in midgut epithelia is primarily mediated by the IMD pathway [37,38,77,78]. Therefore, the characterization of the activation of the Toll pathway in the brain and its involvement in immune reactions in the CNS deserves further investigation in conditions such as disease, injury and infection, as it appears that different innate immune pathways are required in different tissues and biological contexts.

##### IMD Pathway

The second NF-κB pathway in *Drosophila*, the IMD pathway, also contributes to neuropathology in fly models of neurodegenerative disease such as Ataxia telangiectasia (A-T) [79,80], retinal degeneration [55], as well as brain-specific bacterial injection [54] and traumatic brain injury [73,81]. Genetic evidence supports the involvement of the IMD pathway components Dredd (caspase), Kenny (IkB kinase (IKK) protein) and Relish (transcription factor) in retinal degeneration. However, the authors propose a non-canonical activation of the pathway in this context as the adaptor proteins Imd and dFADD are not required for degeneration [55]. Studies done in another fly model of human neurodegenerative disease, A-T, demonstrate that Relish is necessary for neurodegeneration and that overexpression of a constitutively active form of Relish (Rel-D) specifically in glial cells leads to neurodegeneration in this model [80]. Antimicrobial peptide reporter gene expression co-localizes with glial cells in A-T flies, uncovering for a first time that glial cells within the fly brain are able to produce these molecules [79].

Brain-specific injection of a mix of *Escherichia coli* (Gram-negative bacteria) and *Micrococcus luteus* (Gram-positive bacteria) leads to local activation of the IMD pathway reporter gene *Attacin::GFP* [54]. In addition to the previous observation that AMPs localize in glial cells when the gene *A-T mutated* (*ATM*) is mutated, this observation is an indication that the fly brain responds to microbial stimuli and is able to activate protective innate immune mechanisms. In this context, even though flies survive the microbial challenge, they exhibit long term sequelae as observed in mammalian models, which are characterized by the progressive, age-dependent neurodegeneration and reduction in locomotor activity [54]. These brain infection experiments indicate that flies can be used as a model to study brain immunity to infection as well as the long-term consequences of such challenges. Moreover, glia- or neuron-specific knockdown of the transcription factor Relish suppresses the age-dependent neurodegeneration that occurs following bacterial injection, placing the overactive IMD pathway as central contributor to neurodegeneration. Increase in expression of AMP genes, which are normally downstream targets of IMD signaling, is found in fly heads and brain tissue from flies subjected to TBI [73,74], flies exposed to radiation [75] as well as in aged flies [82].

A recent study in which adult flies were subjected to infection with the neurotropic ZIKA virus (ZIKV) demonstrates activation of the IMD pathway in the brain [83]. In this model, *Diptericin* (downstream of IMD signaling), but not *Drosomycin* (downstream of Toll signaling), is upregulated in fly heads that have been infected with the virus, a change that is not observed in *relish* null mutants. Therefore, the *Drosophila* IMD pathway in the brain appears to be required to restrict ZIKV infection in this tissue [83].

#### 2.1.2. Autophagy

Autophagy is an evolutionarily conserved, homeostatic mechanism that promotes degradation of waste components from the cytoplasm in acidic lysosome compartments [84]. This process is particularly important for maintaining optimal health of the cells in the nervous system by assuring degradation of toxic molecules and damaged organelles in post-mitotic neurons [85,86,87,88,89]. Autophagy can also be induced following infection with pathogens, including viruses, contributing to some extent, to antiviral defenses [90,91] (Figure 1). In *Drosophila*, ZIKV infection induces antiviral autophagy in the brain as illustrated by the increased appearance of fluorescent foci from the autophagy reporter construct *mCherry-Atg8*, a process that is Relish-dependent [83]. The fly ortholog of the mammalian polyubiquitin-binding scaffold protein p62, the autophagy cargo receptor Ref(2)P, is a known restriction factor of *Drosophila* Sigma virus (DMelSV), naturally occurring pathogen of flies [92,93]. DMelSV infects the fly brain rendering flies more sensitive to exposure of CO_2_ [94]. Ref(2)P is also antiviral against ZIKV in the brain, because its knockdown leads to increased rate of infection with ZIKV of fly heads [83]. Although the role of autophagy in the fly brain is well established for the maintenance of neuronal homeostasis and the prevention of neurodegeneration [87], more needs to be learned about its antiviral and general anti-pathogenic roles in the context of brain infections. Flies represent an excellent system to address these studies and in the future are likely to provide valuable insights for these disease conditions as well.

### 2.2. Phagocytosis

Phagocytosis is an important defense mechanism that has been conserved during evolution and is a powerful way for *Drosophila* to eliminate apoptotic bodies or bacterial infection [42]. A subtype of *Drosophila* hemocytes, called plasmatocytes, can internalize a large variety of particles, including bacteria, within minutes [25,95]. This complex cellular process is initiated by the recognition of the particle to be ingested, followed by cytoskeletal remodeling and signaling events leading to the engulfment and destruction of the particle. In flies, several phagocytic recognition receptors have been identified, among which are the EGF-like repeat-containing proteins Nimrod C1, Eater, and Draper as well as the CD36-like molecule Croquemort and the Scavenger Receptor SR-CI. These receptors play a role in the recognition and uptake of Gram-positive and Gram-negative bacteria [96,97,98,99,100].

Phagocytosis also takes place in the *Drosophila* CNS. This process is particularly important in the developing brain where it ensures the removal of excess apoptotic neurons, pruned neuronal branches, or synapses [101]. In the adult, brain phagocytosis also plays a neuroprotective role by removing axonal debris following axonal injury, during aging or in a fly model of AD [53,101,102,103,104,105]. The primary cell type that mediates phagocytosis following axonal injury is escheating glia, which exert functions similar to those of mammalian microglia and astrocytes [52,101]. The phagocytic receptor Draper is required for glial phagocytosis following axonal injury and its downstream signaling depends on phosphorylation by the *Drosophila* tyrosine kinase Src42a, the non-receptor tyrosine kinase Shark, interactions with the PTB domain protein dCed-6 and the GTPase Rac1, resulting in actin-dependent cytoskeleton reorganization and engulfment [106,107]. Draper signaling following injury results in activation of the transcription factor STAT92E, which mediates transcription of additional *draper* mRNA following injury to enhance phagocytosis of axonal debris [106] (Figure 1). *Draper* mutants exhibit short lifespan [103] and age-dependent neurodegeneration due to defects in phagosome maturation and inefficient removal of apoptotic corpses generated during development [105].

Retinophilin also known as Undertaker, a membrane occupational and recognition nexus (MORN)-repeat containing protein, is involved in regulating cytoplasmic calcium levels preceding phagocytosis in embryonic macrophages [108]. Moreover, Retinophillin protects axons in the *Drosophila* brain from degeneration in the presence of taxol [104]. Fragile X mental retardation 1 (FMR1) is an evolutionary conserved RNA binding protein implicated in autism. *Drosophila FMR1* mutants exhibit defective phagocytosis in the adult brain, leading to delayed axonal clearance and delayed recruitment of phagocytic glia to the wounded site following axonal injury [109]. Together, these studies implicate phagocytosis as another neuroprotective mechanism in the *Drosophila* brain.

Phagocytosis and autophagy were previously thought to be distinct cellular pathways. However, many autophagy proteins were found to participate in the later stages of phagocytosis. Following infection, both phagosomes and autophagosomes eventually fuse with lysosomes to degrade ingested pathogens [110,111]. Neurodegeneration and corpse accumulation in *draper* mutants can be rescued by glia-specific inhibition of autophagy initiation in *Drosophila* through mechanism involving *Atg1*, *Atg12* and *Atg6*, but not *Agt7*, *Atg8*, and *Ref(2)P*. This is interesting because infection with ZIKV that induces antiviral autophagy in neurons depends on *Atg5*, *Atg7*, *Atg8*, and *Ref(2)P*, while both neuronal and glial depletion of *Atg8* leads to increase in ZIKV replication [83]. As phagocytosis and autophagy are conserved mechanisms linked to each other, it would be of interest to investigate their relationship during brain infection with bacteria and viruses in flies and how their interaction affects pathogen clearance.

The phagocytic capacity of glial cells and the involvement of phagocytic receptors in the context of infections with bacteria and viruses in the brain have not been addressed so far. As phagocytosis by hemocytes following infection with bacteria and some viruses is a protective cellular mechanism that clears infection and apoptotic cells in a Draper-dependent manner [91,112,113], it would be of great interest to examine how this process contributes to the clearance of bacterial and virus infections in the *Drosophila* CNS. With the excellent cell biology and genetic tools that exist in *Drosophila*, future efforts should be combined to address this question in CNS infectious contexts as well.

### 2.3. RNA Interference: Transposon and Virus Control in the Brain

RNA interference (RNAi), and more specifically the small interfering RNA (siRNA) pathway, is a major defense mechanism against viruses and mobile genetic elements in somatic tissues in *Drosophila* [114,115]. This evolutionary conserved pathway relies on the sensing of double-stranded RNA (dsRNA) from exogenous or endogenous sources (e.g., products of viral genome replication or transposons, natural antisense transcripts (NATs) or structured RNAs), which are processed into siRNAs of 21 nt of length by the enzyme Dicer-2 in the cytoplasm. Subsequently, Argonaute-2 (AGO2), which is the catalytic subunit of the RNA-induced silencing complex (RISC), interacts with siRNAs and uses them as guides to identify complementary sequences in target RNAs and induce their degradation [116,117,118,119,120] (Figure 1).

RNAi appears to function as an antiviral mechanism in the brain following infection with the alphavirus Sindbis virus (SINV). This role is illustrated by the increase in SINV virus load in heads of *AGO2* mutants, suggesting that antiviral RNAi is effectively fighting viruses in the *Drosophila* CNS [83]. Interestingly, protection against ZIKV, which has a genome that is also a positive-stranded RNA molecule, is not dependent on RNAi in the brain [83]. However, another study found that *Dicer-2* mutants have increased sensitivity to ZIKV infection and exhibit higher viral loads [121].

Another line of evidence implicates the RNAi mechanism in neuroprotection in the context of aging. In *Drosophila*, transposable elements (TE), which are genetic mobile elements having the capacity to replicate and insert into a new location in the genome, are highly abundant in the aging brain. Transcription of the retrotransposable elements *R2* and *gypsy*, is elevated in aged flies compared to young animals. AGO2 contributes to the control of TE during aging, as *AGO2* mutants exhibit accelerated rates of increase in *R2* and *gypsy* expression in the brain in comparison with wild type controls [122]. About 30% of the *Drosophila* genome is composed of TE, and TE mobilization can represent a source of DNA damage and genetic instability [122]. Derepression of TE has been reported in human neurodegenerative diseases [123,124] and in a fly model of ALS [125], implicating RNAi as a neuroprotective mechanism in flies.

## 3. Regulation of Innate Immunity in the Brain

Unrestrained immune and inflammatory responses can have harmful consequences that can result in tissue damage [3,126]. In *Drosophila*, the IMD pathway is negatively regulated at almost every step of the signaling cascade [127] and loss of intracellular negative regulators such as Pirk, Dnr1, Trabid and Transglutaminase (Tg) (Figure 1) results in early onset neurodegeneration and shorter lifespan pointing to a neuroprotective role for these factors with age [54,82]. Moreover, expression of genes encoding for several intracellular negative regulators of IMD signaling in the head decreases with age and is paralleled by an increase in expression of genes encoding AMPs [82]. This age-dependent increase of downstream targets of the NF-κB transcription factor Relish in heads and brains indicates that flies, similar to mammals, develop inflammaging. The upstream cellular processes and factors that contribute to inflammaging as well as the effects of inflammaging on various disease states are not fully understood, and flies can therefore represent an excellent model to decipher the complex regulation of innate immune reactions in this context.

Albeit to a lesser extent, Toll pathway-dependent AMP expression also increases with age. mRNA of the NF-κB transcription factor *Dif* is significantly upregulated in heads of older flies. However, non-significant change is observed in *Cactus* expression [82]. Both Dif and Cactus proteins are expressed in the *Drosophila* brain [70], although it is not exactly known how Toll signaling activity is regulated with age.

In a *Drosophila* model of Poly-Glutamine (Poly-Q)-mediated neurodegeneration, the transcriptional co-activator of the Hippo pathway, Yorkie (Yki), negatively regulates both Toll and IMD pathways in the fly eye through *cactus* and *relish*, respectively. (Poly-Q) aggregates, which inhibit Yorkie, also lead to an increase in AMP expression and neurodegeneration. Overexpression of Yki in photoreceptor cells in the eye reduces Poly-Q-induced AMP expression and suppresses neurodegeneration [128].

Autophagy inhibition is associated with premature aging and the development of neurodegenerative phenotypes [129,130]. It is also known that the expression of autophagy genes decreases with age in neural tissue in flies [131]. It would be therefore interesting to determine whether it is in the aging brain that IMD/Relish signaling is altered, and to what extent autophagy functions as an antimicrobial mechanism able to restrict pathogens. Similarly, age-dependent decline in the phagocytic capacity of glial cells to remove axonal debris is observed in flies [102] and it would be of great interest to gain further insights about the role phagocytosis plays following infection of the aged brain.

## 4. Concluding Remarks

Brain disease, injury or infections are often associated with deleterious outcomes for affected individuals. Excessive inflammatory responses are frequently observed in neurodegenerative diseases and are thought to underlie the onset and progression of such disorders. Despite advances in vaccinations and the development of improved antibiotic treatments, CNS infections continue to be a source of significant morbidity and mortality worldwide. The fruit fly *Drosophila* offers many experimental advantages and has greatly contributed to discoveries about innate immune signaling activation and the mechanism underlying nervous system development and disease. We are just starting to learn about the brain-specific innate immune mechanisms in flies. How exactly neuro-inflammatory reactions exert neurotoxic effects remains to be determined. Despite a handful of reports on brain injury and infection, our understanding of the protective antimicrobial mechanisms in the *Drosophila* brain remains limited. Additionally, studying the long-term consequences of brain infections deserves further attention as neuropsychiatric disorders can develop following CNS infection. In the future, flies should continue to serve as model to investigate innate immune mechanisms in the brain including their antimicrobial and neuroprotective roles in this tissue.
